# Human–AI interaction in a cancer-enriched double-reading breast screening cohort: diagnostic accuracy and second-reader behavior

**DOI:** 10.1186/s40644-026-00995-0

**Published:** 2026-01-24

**Authors:** Eloïse Sossavi, Mickaël Tardy, Florie Hurstel, Jean Schwartz, Antoine Wackenthaler, Claire Harter, Julien Uttner, Mélanie Mollion, Marie-Françoise Bretz, Sébastien Molière

**Affiliations:** 1 Radiology Department, Hautepierre Hospital, Strasbourg University Hospital, 1 Avenue Molière, Strasbourg, 67098 France; 2 Hera-MI SAS, Nantes, France; 3 Ecole Centrale de Nantes, Nantes, France; 4 Hôpitaux Civils de Colmar, Colmar, France; 5 Institut de Cancérologie Strasbourg Europe (ICANS), Strasbourg, France; 6 Institut de Génétique et de Biologie Moléculaire et Cellulaire (IGBMC), Strasbourg University, Strasbourg, France

## Abstract

**Rationale and objectives:**

To evaluate the impact of deploying AI as the first reader (R1) in a double-reading breast-screening workflow and to characterize second-reader (R2) behavior—including the effect of disclosing whether R1 was AI or human.

**Materials and methods:**

This retrospective study used a cancer-enriched cohort of 220 women (95 cancers), with prevalence-weighted analyses performed to approximate population screening metrics. Five radiologists and one commercially available AI (Breast-SlimView^®^, Hera-MI) each served as R1; four radiologists served as R2. For each R2, cases were randomized 1:1 to AI-first versus human-first and, independently, to disclosure versus concealment of R1 identity. R2 could validate, dismiss, or add annotations. The primary endpoint was final decision correctness by breast. We used GEE logistic regression to estimate the overall effect of using AI as the first reader and to isolate second-reader behavior independently of first-reader accuracy.

**Results:**

At the prespecified R1 operating point, AI had sensitivity/specificity/accuracy of 85.2%/79.5%/80.8% versus 84.3%/84.5%/85.0% for human R1s; crude final accuracy was lower for AI-first. At 0.6% prevalence, AI-first yielded higher recalls (20.8% vs. 16.8%) with slightly lower PPV (2.7% vs. 3.0%). Conditioning on R1 correctness, R2s were approximately twice more likely to overturn an incorrect AI-initiated opinion than an incorrect human-initiated one (OR ≈ 2.05, *p* < 0.001). Disclosure that R1 was AI increased R2 corrections (from 13.6% to 19.1%, *p* = 0.029). Thirteen AI-true-positive cues were dismissed by R2.

**Conclusions:**

At this operating point, AI-first reduced crude accuracy due to lower specificity, yet reader-behavior analyses indicate greater scrutiny of AI-initiated opinions. Protocol, threshold, and user-interface choices may raise specificity while preserving beneficial human–AI dynamics.

**Supplementary Information:**

The online version contains supplementary material available at 10.1186/s40644-026-00995-0.

## Highlights

In a double-reading breast screening simulated workflow with both human and AI as a first reader, AI-first final accuracy was lower than human-first (80.8% vs. 85.0%).

At the prespecified R1 threshold (matching average human R1 sensitivity), AI had lower specificity, yielding more false positives.

Second readers overturned AI-initiated errors more often than human-initiated (controlled-direct effect odd ratio ≈ 2.1).

Disclosing AI identity increased R2 correction (from 13.6% to 19.1%; *p* = 0.029).

**Supplementary Information:**

The online version contains supplementary material available at 10.1186/s40644-026-00995-0.

## Introduction

Double reading of mammograms is an established cornerstone of breast cancer screening, enhancing diagnostic accuracy and reducing false-negative diagnoses [1, 2]. More recently, the integration of artificial intelligence (AI) into clinical practice has provided radiologists with powerful decision support tools, and AI-based systems have demonstrated promising standalone performance [3].

Yet when AI is integrated into real-world workflows—whether as a triage gate-keeper or as an independent “second reader” [4]—the downstream effects on recall rates, arbitration volume and net human reads depend critically on how often AI and humans disagree [5, 6]. Retrospective simulations risk underestimating this arbitration burden, and real data on AI–human discordance remain scarce.

In this study, we evaluated the effect of deploying AI as the first reader in an unblinded, double-reading breast‐cancer screening workflow. Our goals were twofold: first, to quantify AI–human discordance and overall screening performance when every AI‐read case is still subject to full human review; and second, to uncover any reader biases that emerge when the second reader (R2) knows whether the first read was performed by AI or by a fellow radiologist.

To this end, we conducted a retrospective, multi-reader study to quantify (i) the total effect of assigning AI as the first reader on screening performance (final correctness by breast), compared with human-first, and (ii) a controlled direct effect that isolates second-reader behavior by conditioning on whether the first read was correct. We also tested whether randomized disclosure of the first reader’s identity (AI vs. human) changes the second reader’s propensity to overturn incorrect first-reader opinions and the resulting AI–human discordance.

By grounding our analysis in actual consensus decisions—rather than in simulated arbitration algorithms—we deliver the first detailed characterization of how an AI-first model transforms both diagnostic accuracy and error-correction dynamics within a real-world breast-screening program.

## Methods

### Study design

This retrospective multi-reader (9 readers + 1 AI system) study simulates a double-reading screening process to evaluate the impact of incorporating AI as the first reader (R1) on the performance of the human second reader (R2). In addition, we assess the influence of disclosing versus concealing the identity of the first reader (i.e., AI versus radiologist) on diagnostic decision-making.

### Database

We analyzed a cancer-enriched cohort of 220 women referred to our breast cancer center for screening. Eligible patients had two-view mammograms (cranio-caudal and mediolateral oblique) for each breast. Studies involving tomosynthesis were excluded, as were patients with a history of breast surgery or malignancy, the presence of breast implants, or mammograms deemed technically insufficient for analysis.

### Reference standard

The reference standard was established using a normal two-year follow-up for non-cancer cases and pathology confirmation for cancer cases. Lesion segmentation on the mammograms was performed in consensus by an expert radiologist (15 years of experience) and a subspecialty radiologist (5 years of experience), with supplemental use of MRI and large-slice pathology to enhance accuracy and consistency.

### Standard-of-care context

In routine breast cancer screening, double reading typically involves two independent radiologist interpretations followed by arbitration in case of disagreement, with substantial variability across programs regarding arbitration procedures and information sharing. The present study used a deliberately transparent workflow with systematic second-reader review and access to first-reader annotations to specifically investigate second-reader behavior and human–AI interaction.

### Double reading flowchart

**Fig. 1:**
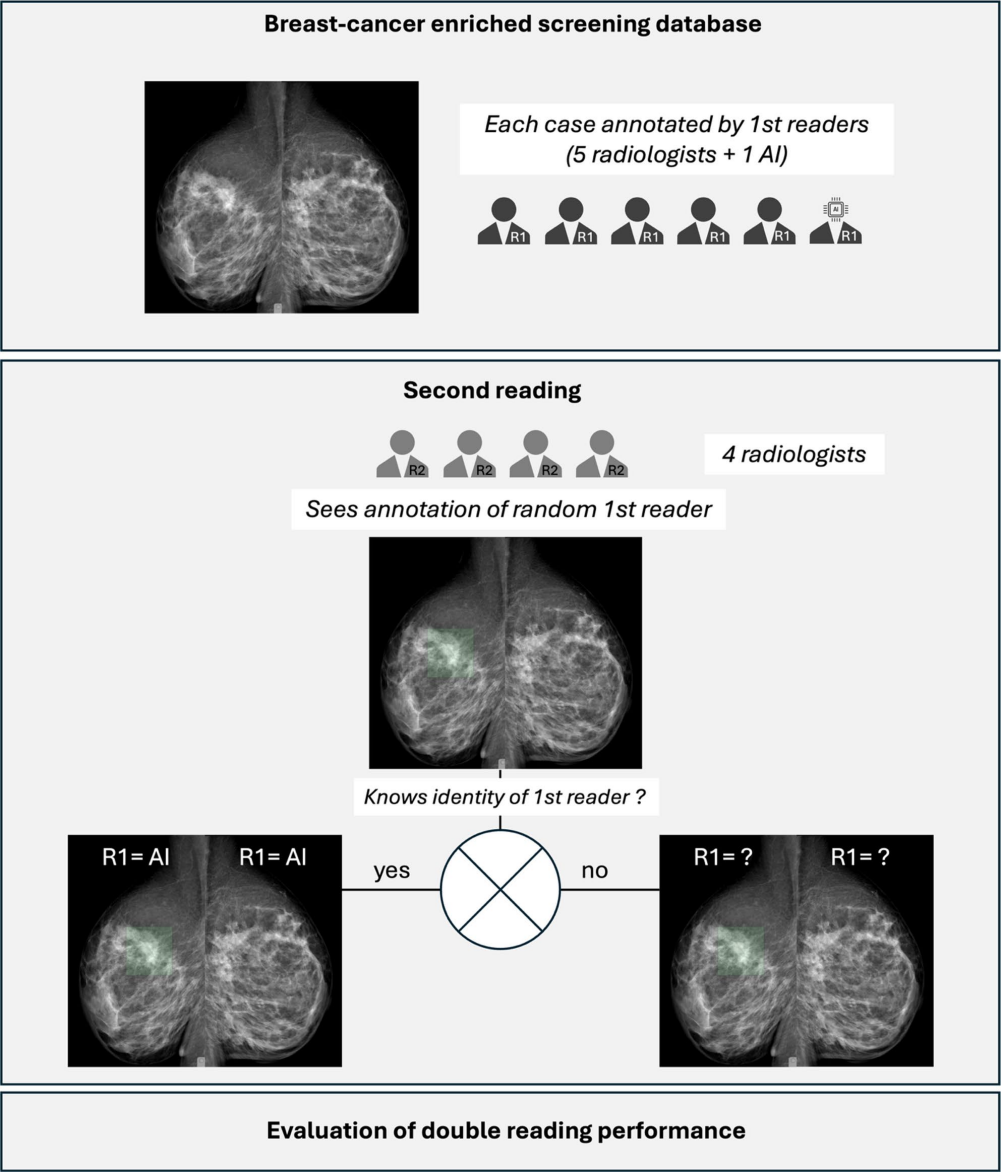
General flowchart of the study

Figure 1 illustrates the double-reading workflow employed in this study.

**First Reading**. Independent annotations of suspicious lesions were performed on all available mammographic views by five radiologists with varying levels of experience (25, 15, 5, 2, and 1 years) and by the AI model.

**Second Reading**. Four different radiologists with 18, 5, 4, and 3 years of experience independently reviewed the mammograms from all 220 women. For half of the cases, the second readers were provided with the first reader’s annotations while remaining blinded to the source; for the other half, the identity of the first reader (AI or human) was disclosed. During review, the second readers had the option to validate, dismiss, or add new annotations. All second readers were provided with the AI system’s theoretical diagnostic performance; of the four, one had two years of hands-on experience with the tool, one had under one year, and two had no prior professional exposure to it.

**Allocation & crossover**. For each of the four R2 radiologists, all 220 cases were read once, in a fixed reading order specific to that reader. Two orthogonal 1:1 randomizations were generated a priori for each R2 list: (i) R1 type (AI-first vs. human-first; when human-first was assigned, the specific R1 was sampled uniformly from the five radiologists), and (ii) disclosure (R1 identity disclosed vs. concealed). Randomization was implemented using Python (NumPy random module) with a fixed random seed to ensure reproducibility. Randomization used permuted blocks within strata defined by cancer status (present/absent) and ACR density (American College of Radiology; A/B vs. C/D) to ensure balance. Thus, each case was read once by each R2 under a preassigned combination of R1 type and disclosure, yielding an approximately 1:1 distribution for both factors within every strate and reader. Second readers were not informed of the specific behavioral hypotheses under investigation, and no feedback was provided during or after the reading sessions.

**Reader calibration.** Before data collection, all R2s completed a 30-min calibration reviewing 10 sample cases (not used in the study), aligned on use of the annotation tool, and reviewed a one-page summary of AI standalone performance and common failure modes. No feedback was provided during the study.

### AI model

The AI model used in this study (Breast-SlimView, Hera-MI, Rennes, France) is a commercially-available U-Net deep neural network specifically trained to segment suspicious lesions according to the American College of Radiology Breast Imaging Reporting and Data System (BI-RADS) classification. The AI’s positivity threshold was calibrated specifically for this study, to match the average sensitivity of human first readers. To maintain blinding and prevent the second readers from distinguishing between AI- and radiologist-generated annotations, all annotations were displayed solely as bounding boxes centered on the detected anomalies.

### Outcome and covariable definitions

The primary outcome was based on the final screening decision by breast. To assess factors influencing this outcome, we employed a generalized estimating equations (GEE) logistic regression model. The model included the following predictors: breast density, R1/R2 reader pair type (AI-first vs. human-first), disclosure of R1’s identity, and R1 correctness. The GEE approach was used to account for the within-patient correlation, with an independence working correlation structure and robust standard errors. This model allowed to assess the independent contributions of the predictors while adjusting for repeated measures from the same patient.

### Retrospective labelling of AI annotations

All annotations by the AI were reviewed in consensus by two radiologists (ES and SM) to determine the plausible anomaly captured by the AI-generated bounding boxes. Lesions were categorized as masses, distortions, areas of focally increased density, microcalcifications, macrocalcifications, or a combination. If no visible anomaly was identified within the bounding box by human readers, the region was labeled as “normal breast parenchyma.” This post-hoc categorisation of each AI box was performed offline after the study solely to analyse error types and did not alter any image shown to second readers.

### Statistics

Descriptive statistics were calculated for all study variables. Continuous variables were summarized as means ± standard deviations or medians with interquartile ranges, as appropriate. Categorical variables were presented as frequencies and percentages.

To assess overall diagnostic performance, we calculated standard performance metrics (accuracy, sensitivity, and specificity) for the final screening decision—defined as correct when the second reader’s outcome was a true positive or true negative—against the reference standard. These metrics were also computed separately for cases where the first reader was a radiologist (human-first) versus AI (AI-first). To approximate population screening (at a 0.6% prevalence), we applied inverse-probability weights at the breast level—w_cancer = 0.006/π and w_non-cancer = 0.994/(1 − π), where π is the observed prevalence—and computed weighted confusion matrices to derive PPV/NPV, expected recall rate, and discordance rate between R1 and R2 per 1,000 screens.

Inter-reader concordance between the first and second readings was evaluated by contingency tables and calculating the overall agreement rate as well as Cohen’s kappa statistic. McNemar’s test was used to compare paired binary outcomes between the first and second readers, particularly to assess the extent of error correction. Two-proportion z-tests were applied to assess differences in correction rates for specific lesion types identified by AI.

To investigate the independent effects of the study variables on the final screening decision, we employed logistic regression models. Conceptually, two complementary questions were addressed. First, the total effect (TE) asks: “If a human first reader is replaced by AI, what happens to final screening accuracy?” This total effect combines two mechanisms: differences in first-reader diagnostic performance and differences in how second readers respond to AI versus human initial opinions. Second, the controlled direct effect (CDE) asks: “When the first read is equally correct or equally incorrect, does the second reader respond differently depending on whether that initial opinion originated from AI or from a human reader?” By holding first-reader correctness constant, this effect isolates second-reader behavior independently of AI diagnostic accuracy.

Accordingly, we report the total effect of assigning AI as the first reader (AI-first) versus a radiologist (human-first) on the binary endpoint of final correctness (per-breast), via a crude logistic regression model with AI-first as the sole exposure as well as a case-mix–adjusted logistic regression adding breast density and reader fixed effects. To characterise second-reader behavior, we also report a controlled direct effect of AI-first holding constant the correctness of R1 (mediator), which captures how R2 acts when presented with a correct vs. incorrect initial opinion, irrespective of whether that opinion came from AI or a human, via a mediator-adjusted model adding R1 correctness [7]. For correlated outcomes we used GEE with an independent working correlation and robust standard error, clustering at the patient level, to account for correlation between breasts and repeated observations within the same patient. As sensitivity analyses we fit (i) mixed-effects logistic models with random intercepts for patient and R2 and (ii) GEE with multi-way clustering (patient and R2).

All analyses were performed in JupyterLab using Python (version 3.11), with key libraries including pandas for data manipulation, NumPy for numerical operations, Statsmodels for regression modeling and GEE analysis, SciPy for statistical tests, and scikit-learn for performance metric computation. Data visualization was carried out using Matplotlib. A two-sided significance level of *p* < 0.05 was considered statistically significant.

## Results

### Population

The study cohort consisted of 220 patients, yielding a total of 1760 double-reading screening observations (average of 8 per patient). Each observation represents a paired assessment by a first reader (R1) and a second reader (R2). Supplemental figure S1 shows the CONSORT-style flow diagram of allocation, randomization, and double-reading workflow. Among the cohort, 95 cases of screening-detected breast cancers were identified in 85 women. Table 1 provides a detailed summary of the population characteristics, including mammographic findings, pathological subtypes, and tumor biomarker profiles.

**Table 1:** All cancers and cancers missed by R1

	All cancers	Cancers missed by ≥ 1 human R1	Cancers missed by AI
	*N* = 95	*N* = 15	*N* = 8
**Breast density**			
A	19% (18)	7% (1)	12% (1)
B	65% (62)	73% (11)	88% (7)
C	14% (13)	20% (3)	0
D	2% (2)	0	0
**Mammography features**			
Mass	72% (68)	47% (7)	88% (7)
Asymmetry / focally increased density	7% (7)	27% (4)	0
Distortion	10% (9)	13% (2)	12% (1)
Microcalcifications	3% (3)	7% (1)	0
Mass with associated microcalcifications	6% (6)	7% (1)	0
**Pathology subtype**			
IDC	74% (70)	47% (7)	75% (6)
ILC	16% (15)	40% (6)	25% (2)
DCIS	6% (6)	13% (2)	0
Other	4% (4)	0	0
**HR positive**	81% (77)	66% (10)	88% (8)
**HER2 positive**	7% (7)	27% (4)	0%
**Triple negative**	6% (6)	7% (1)	12% (1)
**Ki67**	21.7 ± 2.0	21.9 ± 4.1	20 ± 5.7
**Tumor size**	15.7 ± 3.1	16.2 ± 2.6	10.4 ± 2.1

Values are reported as % (n) for categorical variables and mean ± SD for continuous variables. Percentages may not sum to 100% due to rounding

“Missed by ≥ 1 human R1” refers to cancers not identified by at least one of the radiologist first readers. “Missed by AI” refers to cancers not identified by the AI when acting as first reader

Because these categories are not mutually exclusive, a cancer may be missed by both AI and ≥ 1 human R1. HR-positive defined as estrogen receptor and/or progesterone receptor positivity

AI = artificial intelligence; R1 = first reader; IDC = invasive ductal carcinoma; ILC = invasive lobular carcinoma; DCIS = ductal carcinoma in situ; HR = hormone receptor; HER2 = human epidermal growth factor receptor 2

### First reader performances

The AI, when used as a standalone first reader, achieved a sensitivity of 83.6% (95% CI: 76.1–91.2%) and a specificity of 71.5% (95% CI: 66.8–76.3%), resulting in a 74.0% accuracy. In comparison, the average sensitivity of the radiologist first readers was 84.3% (95% CI: 81.0–87.8%) with a mean specificity of 84.5% (95% CI: 82.8–86.2%), resulting in a 84.5% average accuracy. Notably, the R1 with the highest specificity demonstrated a sensitivity of 81.5% and a specificity of 96.8%, whereas the reader with the highest sensitivity showed a sensitivity of 89.1% and a specificity of 90.8%. Table 1 provides details on the false negative cases, including mammographic semiology and histological features.

### Double-reading screening performances

The overall accuracy of the final decision (based on the second reader’s outcome) was 82.90%, with a sensitivity of 87.77% and a specificity of 81.61%. When stratified by first reader type, cases where a radiologist served as the first reader achieved an accuracy of 85.00% (Sensitivity: 90.7%, Specificity: 83.6%), while cases where AI served as the first reader reached an accuracy of 80.80% (Sensitivity: 85.2%, Specificity: 79.53%). Table 2 shows the outcomes of R2 based on R1 outcomes. At a prevalence of 0.6%, the estimated recall rate was 20.8% for AI-first versus 16.8% for human-first, corresponding to 208 versus 168 recalls per 1,000 screens, PPV 2.7% vs. 3.0%, and sensitivity 85.2% vs. 90.7%, respectively; see Table S1.

**Table 2:** Contingency table showing agreement between first-reader (R1) and second-reader (R2) screening outcomes (*N* = 1760 double readings)

R1 Outcome \ R2 Outcome	FN	FP	TN	TP
FN	32	0	0	20
FP	0	201	193	0
TN	0	55	943	0
TP	13	0	0	303

TP = true positive; TN = true negative; FP = false positive; FN = false negative, defined relative to the reference standard. R1 = first reader; R2 = second reader

**Fig. 2:**
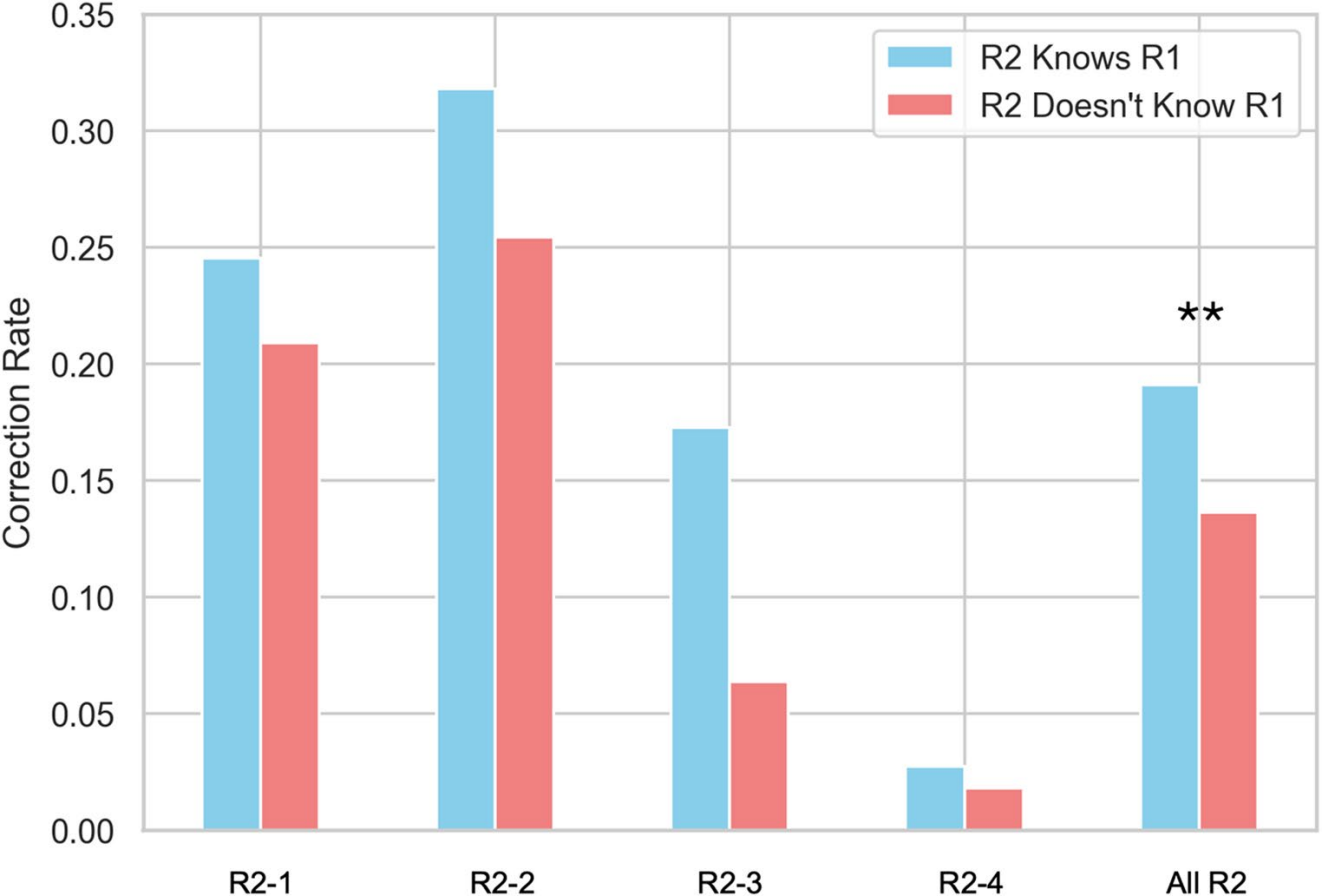
Correction rate by second readers (R2) according to disclosure of first-reader (R1) identity. *All R2* denotes pooled results across all four second readers. Estimates were obtained by aggregating all second-reader interpretations, with statistical analyses accounting for repeated observations per patient. ** indicates a statistically significant difference between disclosure and concealment conditions (*p* < 0.01)

**Fig. 3:**
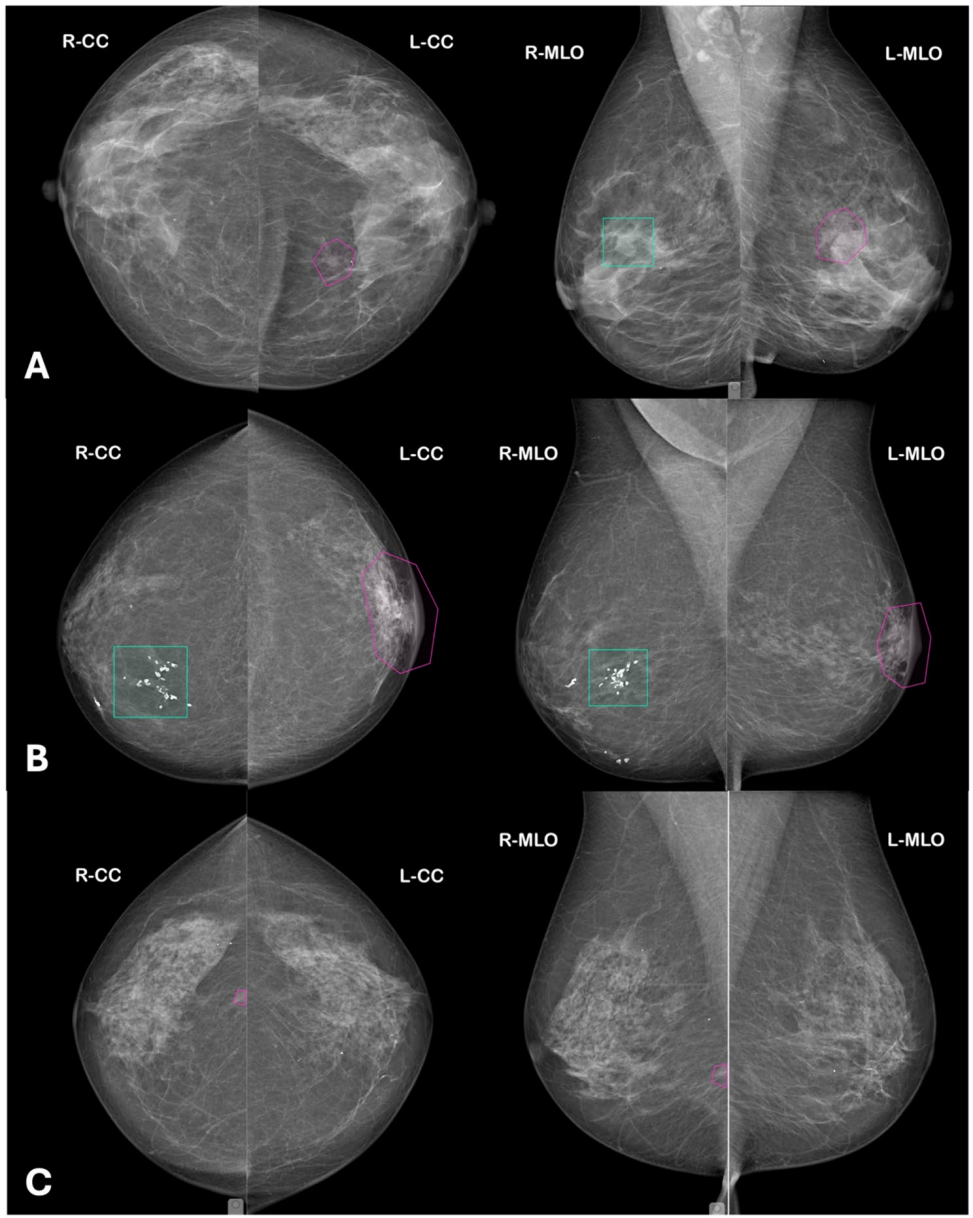
Examples of false-negative cases by the first reader (R1) corrected by the second reader (R2). (**A**) Case with a false-positive annotation by AI as R1 in the right breast and a false-negative lesion in the left breast subsequently identified by R2. (**B**) Case with a false-positive annotation by AI as R1 in the right breast and a distinct false-negative lesion in the left breast corrected by R2. (**C**) Case with a false-negative lesion in the right breast missed by a radiologist as R1 and subsequently identified by R2. In all panels, turquoise bounding boxes indicate the initial annotation by the first reader (either AI or radiologist), and pink polygons indicate corrective annotations made by the second reader

The overall agreement between the first and second readers was 86.6%, with a Cohen’s kappa of 0.706, indicating substantial inter-reader agreement. The agreement was higher in the radiologist-radiologist pair (0.928) versus the AI-radiologist pair (0.803). When the second reader was aware of the first reader’s identity, the correction rate—defined as the proportion of cases in which R2 corrected an error made by R1—improved to 19.09%, compared to 13.64% when this identity was concealed (Z = 2.19, *p* = 0.02875) (Figs. 2 and 3).

Several logistic regression models were used to evaluate the independent effects of reader pair type, disclosure of the first reader’s identity, breast density, and first reader correctness on the final decision (Table 3).

**Table 3:** Variables influencing R2 accuracy

	OR	95% CI	*p*-value
**Total effect (non-adjusted)**			
Intercept	5.63	4.54–5.97	< 0.0001
AI-first (vs. human-first)	0.75	0.58–0.97	0.028
**Total effect (adjusted)**			
Intercept	5.96	3.69–9.67	< 0.0001
AI-first (vs. human-first)	0.76	0.59–0.98	0.033
Disclosure of R1 identity	1.42	1.14–1.78	0.002
Breast density (B vs. A)	0.81	0.51–1.28	0.36
Breast density (C vs. A)	0.71	0.40–1.27	0.25
Breast density (D vs. A)	0.68	0.35–1.32	0.25
**Controlled-direct effect**			
Intercept	0.35	0.19–0.64	< 0.0001
AI-first (vs. human-first)	2.14	1.45–3.15	< 0.001
Disclosure of R1 identity	1.58	1.17–2.14	< 0.0001
Correctness of R1 annotations	51.19	31.26–83.80	< 0.0001
Breast density (B vs. A)	2.79	1.80–4.32	< 0.0001
Breast density (C vs. A)	0.40	0.25–0.64	0.0001
Breast density (D vs. A)	0.48	0.30–0.77	0.0023

OR = odd ratio; R1 = first reader

In unadjusted analyse, AI-first had lower final accuracy than human-first (total effect), driven by AI’s lower R1 specificity. In the mediator-adjusted model that conditions on R1 correctness, AI-first showed higher odds of a correct final decision (OR = 2.14, 95%CI 1.45–3.15), indicating that R2s corrected AI-initiated errors more efficiently than human-initiated errors when presented with equivalently right or wrong first reads (controlled-direct effect). As shown in subsequent analyses, this behavioral effect was primarily driven by correction of AI false positives. Disclosure of the first reader’s identity further improved performance (OR = 1.58, 95%CI 1.17–2.14).

### Review of AI False positives and R2 correction rate

**Fig. 4:**
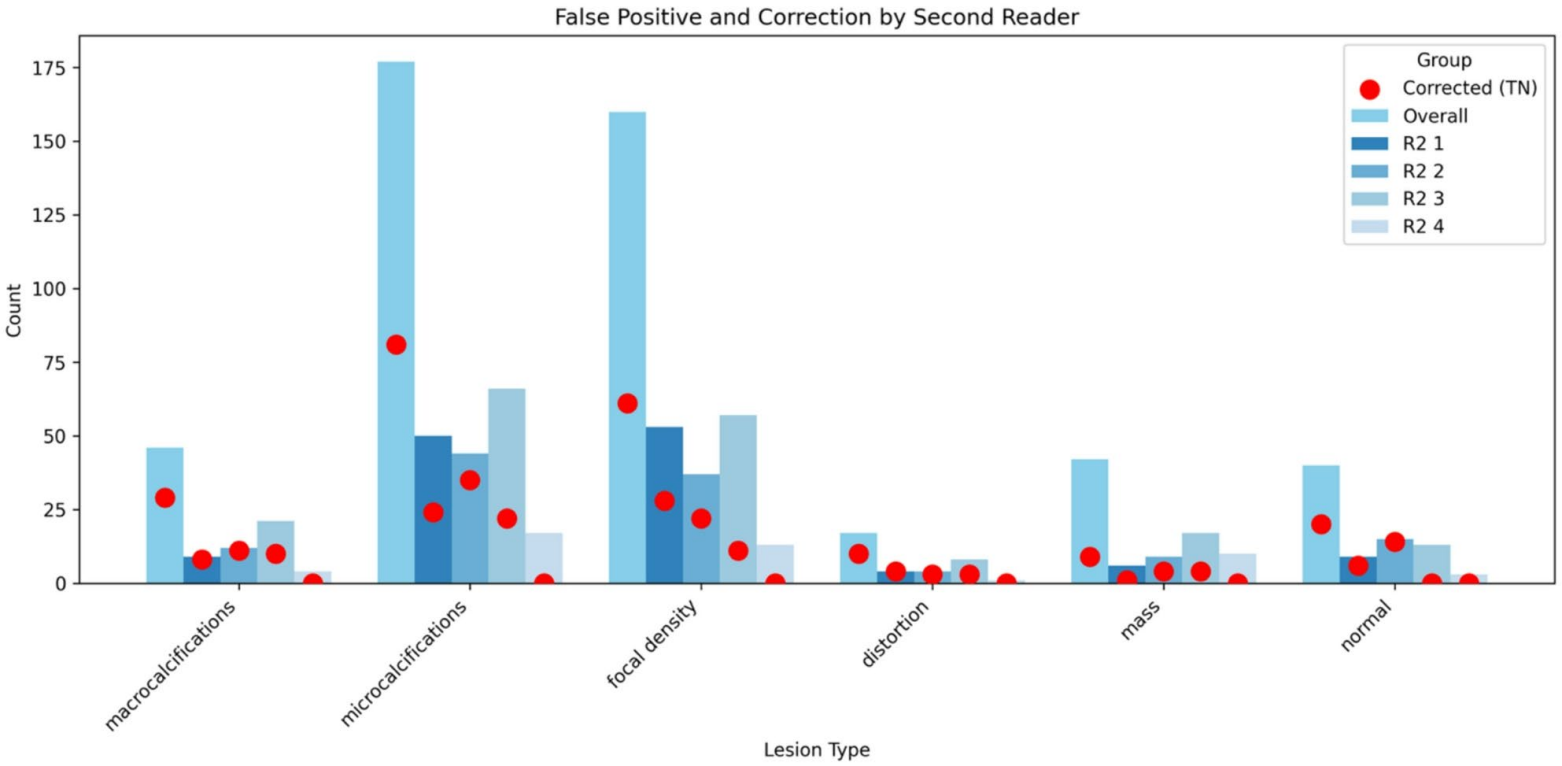
AI false positives by lesion type and correction by second reader (R2)

All AI-detected lesions—whether true positives or false positives—were subsequently reviewed by two expert radiologists. Among the false positives, microcalcifications and focal areas of increased density together accounted for roughly two-thirds of cases (Table 4). When we looked at how often R2 corrected these AI errors (Fig. 4), we found that: benign macrocalcifications were corrected in 63% of cases (29 out of 46), benign microcalcifications were corrected in 46% of cases (81 out of 177), benign masses were corrected in only 21% of cases (9 out of 42).

**Table 4:** Mammography features of AI-detected lesions

	All detected lesions	False positive lesions	*p*
	*N* = 420	*N* = 304	
Mass	121 (28.8%)	29 (9.5%)	< 0.0001
Asymmetry / focally increased density	102 (24.3%)	99 (32.6%)	0.014
Distortion	20 (4.8%)	15 (4.9%)	NS
Microcalcifications	121 (28.8%)	105 (34.5%)	0.005
Macrocalcifications	33 (7.9%)	33 (10.9%)	0.16
Normal breast parenchyma	23 (5.5%)	23 (7.6%)	NS

Values are reported as n (%). Percentages are calculated per column. “False positive lesions” refer to AI-detected findings not corresponding to a malignant lesion according to the reference standard. Lesion categories are identical to those used in Table 1. p values were calculated using two-proportion z-tests to compare the distribution of lesion types between all AI-detected lesions and AI false-positive lesions. AI = artificial intelligence; NS = not significant

### Cancers missed by R2

**Fig. 5:**
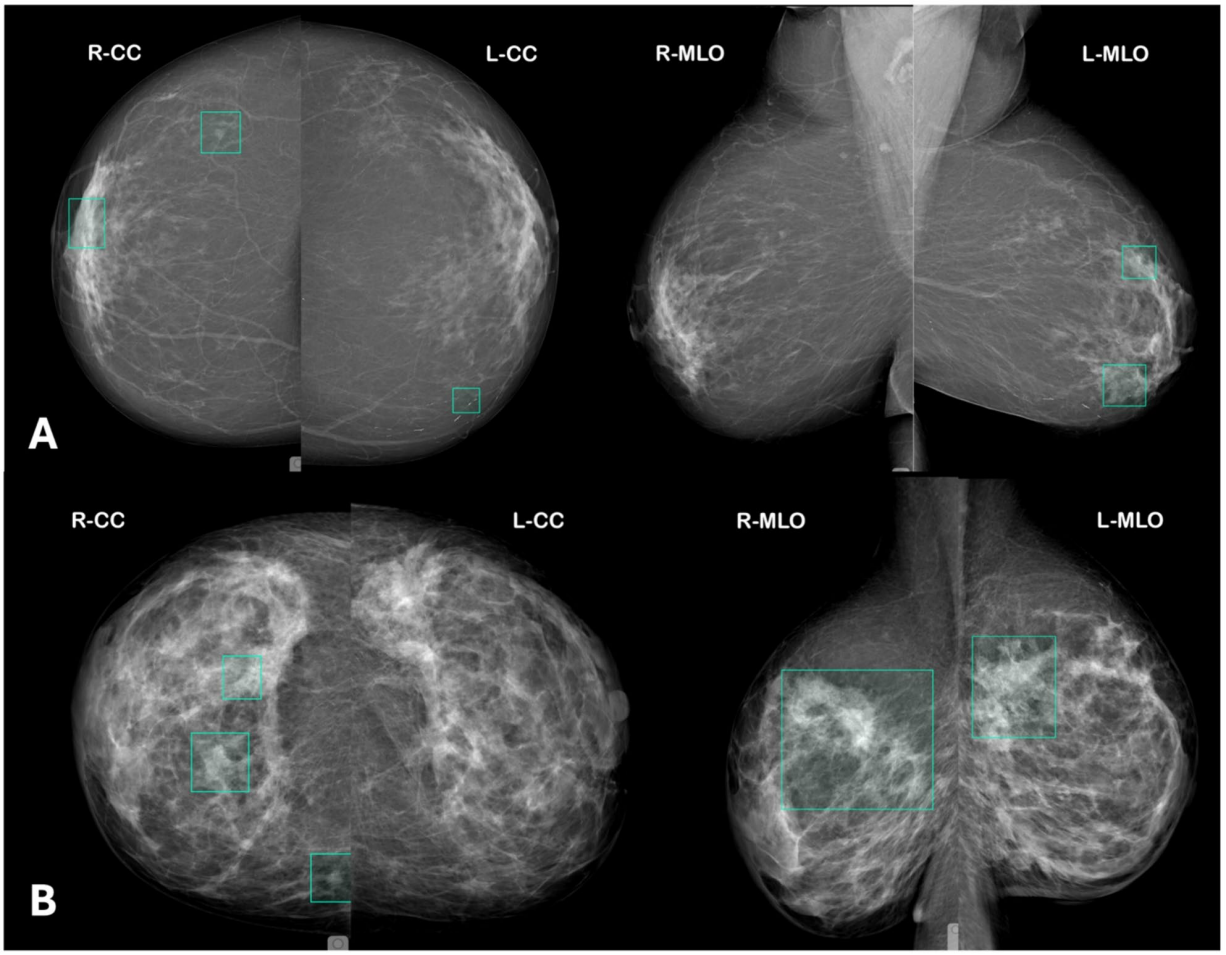
Examples of true-positive lesions detected by AI as first reader (R1) and rejected by the second reader (R2). (**A**) Case in which AI R1 correctly detected a true-positive lesion in the right breast and simultaneously generated a false-positive annotation in the left breast; the true-positive lesion was subsequently dismissed by R2. (**B**) Case in which AI R1 correctly detected true-positive lesions in both breasts, which were subsequently rejected by R2. In all panels, turquoise bounding boxes indicate R1 detections

Out of 1,760 screening observations, 45 (2.6%) were false negatives by R2. In 32 of these cases, both R1 and R2 failed to identify the cancer. The remaining 13 R2 false negatives were AI-flagged true positives that R2 dismissed. Lesions were mostly small masses or subtle asymmetries with a median size of 11 mm (examples can be seen in Fig. 5). False negatives were distributed approximately equally across BI-RADS B and C densities, and none were classic DCIS-type calcification clusters. Seven occurred with disclosure and six with concealment.

## Discussion

AI used as the initial reader matched human sensitivity but, at our prespecified threshold, had lower specificity and therefore lower final accuracy (80.8% vs. 85.0%; total-effect model). To isolate reader behavior independent of the algorithm’s threshold, we estimated a controlled direct effect that conditions on whether the first read was correct. In this analysis, second readers were more likely to overturn an incorrect AI-initiated opinion than an incorrect human-initiated one (≈ 2× higher odds of a correct final decision), while a small number of AI-true-positive cues were dismissed. While this behavioral advantage does not imply net improvement in our study, it may help identify where threshold, user training and user interface tuning can raise specificity while preserving beneficial reader dynamics.

Several real-world implementations have already tested AI as an independent reader in double‐reading protocols. In the *ScreenTrustCAD* non‐inferiority trial, Dembrower et al. replaced the second radiologist with AI, halving independent reads while preserving cancer‐detection (RR ≈ 1.02) but increasing arbitration by 38% [8]. Similarly, Marinovich et al.’s evaluation embedded AI as an independent reader, achieving a 50% reduction in first reads and a 41% drop in total human reads, though arbitration rose by 11% unless thresholds were prospectively tuned [9]. Alternative strategies—such as “either‐positive” recall rules—eliminate arbitration entirely but incur high recall rates and low positive predictive values [10]. Conversely, the MASAI randomized trial’s risk‐allocation triage halved workload without increasing recalls, preserving cancer detection rate (CDR) even in a live service setting [11, 12].

Our unblinded, transparent workflow differs from prior models in that every case—regardless of initial call—undergoes R2 review with full access to R1’s annotations, whether generated by AI or by a radiologist. By eliminating a separate arbitration step, each case that AI “reads” first represents a genuine reduction in R1 workload, rather than a mere shift to a consensus panel. This model parallels selective second-read programs (e.g., only BIRADS-1/2 cases), but applies to all examinations, giving R2 the context needed both to overturn AI false positives and to catch true positives that a blind or AI‐only read might miss. At the same time, presenting R2 with an initial opinion introduces potential anchoring bias—the tendency to rely disproportionately on the first piece of information encountered—and confirmation bias, in which reviewers seek evidence that supports that initial call.

Contrary to typical automation-bias concerns [5, 13], we found that radiologists corrected AI false positives at rates equal to or exceeding their correction of human‐reader errors—particularly when they knew the first reader was AI. Disclosure of AI’s involvement increased R2’s error‐correction rate from 13.6% to 19.1% (*p* = 0.029), suggesting that transparency promotes critical scrutiny. This aligns with prospective diagnostic evidence that AI prompts can influence reader decisions [14]. This “positive bias” likely stems from readers’ calibrated skepticism, informed by an understanding of AI’s comparatively lower specificity.

Nevertheless, a troubling “negative bias” also emerged: in 13 instances where AI correctly flagged cancer, R2 nonetheless dismissed the lesion. All occurred in the AI-first arm (seven with disclosure, six without). Contributing factors may include subtly appearing tumors, cognitive overload from false-positive alerts, or bounding‐box imprecision—either too large or too small to draw attention effectively. These dismissals echo recent prospective evidence that consensus discussions underweight AI flags relative to radiologists, potentially attenuating the benefit of AI decision support [15]. Mitigating these under‐corrections will require targeted interventions: robust training on AI failure modes to sharpen radiologists’ critical faculties, and user‐interface enhancements—such as displaying AI confidence scores, adjusting annotation saliency based on that confidence, or incorporating explainable‐AI heat maps [16].

Interestingly, the blind spots of AI and radiologists differ by lesion appearance and pathology. Cancers missed by either reader tended to be smaller and more often of the lobular subtype, but radiologists were disproportionately likely to miss subtle asymmetries compared with masses, whereas AI rarely failed to detect HER2-positive or DCIS lesions—presumably because it excels at identifying calcification patterns. Second-reader correction rates also varied by lesion type (63% for macrocalcifications, 46% for microcalcifications, but only 21% for masses). Recent studies confirm that AI and radiologists miss different kinds of cancers. van Winkel et al. found AI tends to overlook very small, non-calcified lesions, whereas humans more often miss larger, calcified tumors [17]. Woo et al. reported a 14% AI miss rate—mainly small luminal tumors masked by dense tissue—that experts could still flag on review [18].

### This single-center, cancer-enriched study limits generalisability

First, cancer enrichment may influence reader behavior by increasing vigilance and potentially amplifying both R1–R2 discordance and error-correction rates compared with routine screening. In particular, correction rates observed here should not be interpreted as absolute estimates for population programs. However, because enrichment was uniform across study arms and all comparisons relied on within-reader randomized contrasts, the internal validity of comparative effects—such as differences between AI-first and human-first workflows, or the impact of disclosing R1 identity—is preserved. Prevalence-weighted analyses were therefore provided to contextualize performance metrics at a population level, while behavioral findings are best interpreted as relative effects rather than absolute rates. Also, we did not provide prior mammograms, which are available in most screening settings and likely improve specificity. Results reflect one AI operating threshold; prevalence-reweighted performance are provided to approximate population screening. Finally, while our clustered models account for within-patient and between-reader correlation, the mediator-adjusted analysis (conditioning on R1 correctness) addresses reader behavior rather than net patient impact. Although second readers had varying levels of experience, no strong qualitative differences in the direction of AI-related behavioral effects were observed across readers; however, the study was not powered to formally assess experience-by-AI interactions. Future larger, prospective, multicenter trials featuring readers with varied experience and AI familiarity, with carefully selected AI operating threshold, will be essential to validate and extend these insights.

In conclusion, when integrating a commercially-available AI as a first reader within an unblinded double-reading workflow— the second reader corrected AI false positives at rates equal to or higher than they did for human-first errors—particularly when they were informed that the initial reader was AI. By understanding and mitigating both over- and under-correction biases, we can optimize human–AI collaboration to improve breast cancer screening outcomes.

## Supplementary Information

Below is the link to the electronic supplementary material.

Supplementary Material 1

## Data Availability

Access to data can be granted for legitimate scientific purposes upon reasonable request to the corresponding author and pending additional ethical approval.
