# Supplementary material for: Human–AI interaction in a cancer-enriched double-reading breast screening cohort: diagnostic accuracy and second-reader behavior
Source: Cancer Imaging. 2026 Jan 24;26:29. doi: 10.1186/s40644-026-00995-0 (PMC12910764; doi:10.1186/s40644-026-00995-0)
Supplement: Supplementary file 1 — Supplementary Material 1 [file 40644_2026_995_MOESM1_ESM.docx]

Supplemental data

Supplemental figure S1. CONSORT-style flow diagram of allocation, randomization, and double-reading workflow


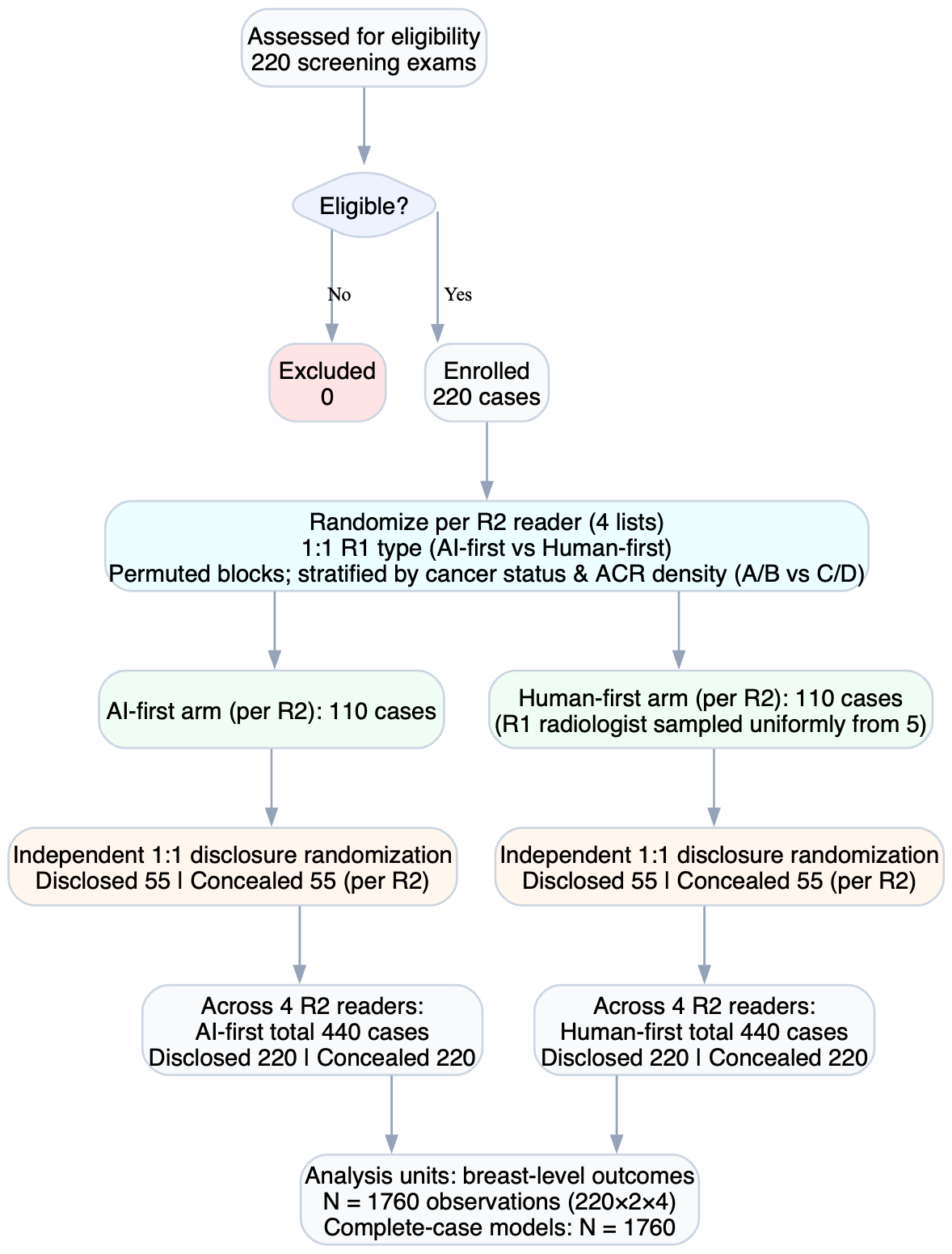


R1 = first reader ; R2 = second reader ; ACR = American College of Radiology

Supplemental table S1. Estimate of population screening performance at a 0.6% prevalence

|  | PPV (%) | NPV (%) | Sensitivity (%) | Specificity (%) | Accuracy (%) | Recall rate (%) | Expected R1-R2 discordance rate (/1000) |
| --- | --- | --- | --- | --- | --- | --- | --- |
| Overall | 2.8 (2.5-3.2) | 99.9 (99.9-99.9) | 87.7 | 81.6 | 81.6 | 18.8 (16.7-20.8) | 147 |
| Human-first | 3.0 (2.8-3.8) | 99.9 (99.9-100) | 90.7 | 83.6 | 83.7 | 16.8 (14.2-19.7) | 73 |
| AI-first | 2.7 (2.1-2.9) | 99.9 (99.8-99.9) | 85.2 | 79.5 | 79.6 | 20.8 (17.8-23.7) | 224 |

Values are reported as point estimates with 95% confidence intervals in parentheses. Estimates were obtained using inverse-probability weighting to extrapolate performance to a population screening prevalence of 0.6%. Confidence intervals were estimated using non-parametric bootstrap resampling with prevalence reweighting applied within each resample. Expected discordance rates are expressed per 1,000 screened examinations.

PPV = positive predictive value; NPV = negative predictive value; R1 = first reader; R2 = second reader.
